# Synthesis and α-Glucosidase Inhibitory Mechanisms of Bis(2,3-dibromo-4,5-dihydroxybenzyl) Ether, a Potential Marine Bromophenol α-Glucosidase Inhibitor

**DOI:** 10.3390/md9091554

**Published:** 2011-09-19

**Authors:** Ming Liu, Wei Zhang, Jianteng Wei, Xiukun Lin

**Affiliations:** 1 Institute of Oceanology, Chinese Academy of Science, 7 Nanhai Rd, Qingdao 266071, China; E-Mails: lmouc@hotmail.com (M.L.); weijt@163.com (J.W.); 2 Southern Research Institute, 2000 9th Avenue South, Birmingham, AL 35205, USA; E-Mail: zhangwee@yahoo.com

**Keywords:** bromophenol, bis(2,3-dibromo-4,5-dihydroxybenzyl) ether, α-glucosidase inhibitor

## Abstract

Bis(2,3-dibromo-4,5-dihydroxybenzyl) ether (BDDE), derived from the marine algae, is a potential α-glucosidase inhibitor for type 2 diabetes treatment. In the present study, a synthetic route was established as a valid approach to obtain BDDE. Fluorescence spectra, circular dichroism spectra and molecular docking methods were employed to elucidate the inhibitory mechanisms of BDDE against α-glucosidase. The results showed that BDDE could be prepared effectively and efficiently with the established synthetic methods. Synthetic BDDE bound with α-glucosidase and induced minor conformational changes of the enzyme. The docking results indicated the interaction between BDDE and α-glucosidase was driven by both hydrophobic forces and hydrogen bonds. The docked BDDE molecule was completely buried in the α-glucosidase binding pocket with part of the molecule reaching the catalytic center and overlapping with the position of glucose, and the rest of the molecule extending towards protein surface. This study provides useful information for the understanding of the BDDE-α-glucosidase interaction and for the development of novel α-glucosidase inhibitors.

## 1. Introduction

α-Glucosidase, is involved in carbohydrate synthesis and breakdown, plays crucial role in diabetes, viral infection and cancer. Due to its diverse bioactivities, α-glucosidase has been considered to be a preferred drug target in the pharmaceutical area and a number of α-glucosidase inhibitors have been discovered and studied. The clinically used anti-diabetic agents acarbose [1], voglibose, and miglitol [2], inhibit α-glucosidase competitively in the brush border of the small intestine, and consequently delay the hydrolysis of carbohydrates and alleviate postprandial hyperglycemia. However, the continuous administration of these agents may cause some adverse effects, such as diarrhoea, abdominal discomfort, flatulence [3–5], and hepatotoxicity [6]. It is therefore still necessary to develop novel α-glucosidase inhibitors. The therapeutic challenge of type 2 diabetes mellitus promotes the search for novel α-glucosidase inhibitors and a number of them have been investigated [7–11].

**Figure 1:**
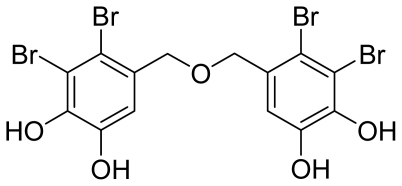
Structure of bis(2,3-dibromo-4,5-dihydroxybenzyl) ether (BDDE).

One such newly reported group of α-glucosidase inhibitors are the marine natural bromophenols [12–15], which are usually isolated from the marine algae. As the α-glucosidase inhibitors, these bromophenols have been reported to inhibit against α-glucosidase with low IC_50_’s (Table 1), and this activity has a close relationship with the Br and phenolic unit in the molecules. One of the most potent bromophenol α-glucosidase inhibitors is bis(2,3-dibromo-4,5-dihydroxybenzyl) ether (BDDE, Figure 1), which competitively inhibits α-glucosidase with an IC_50_ of 0.098 μM [12,13,15]. BDDE has also been reported to possess anticancer [16], and antibacterial activities [17]. The previous promising research results indicate that BDDE represents a novel lead compound for the design of new drugs. However, low content in marine algae as well as the difficulty in extraction and separation have hindered further investigations of BDDE bioactivities. In addition, few efforts have been contributed to explore the interaction between BDDE and α-glucosidase, which is important for dissecting the structure-activity relationship for further optimizations.

In this study, we present a synthetic route that can provide sufficient amounts of BDDE to facilitate bioactivity studies, and we further investigate the mechanisms underlying BDDE inhibition using fluorescence spectra, circular dichroism (CD) spectra, and molecule docking.

## 2. Results and Discussion

### 2.1. Synthesis of BDDE

**Scheme 1:**
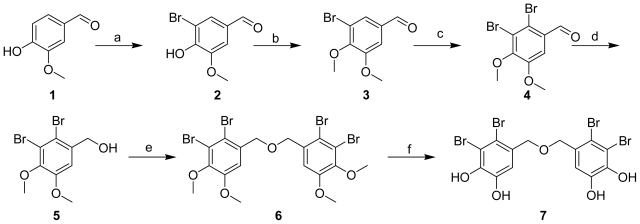
Reagents and Conditions: **a**: AcOH, Br_2_, r.t. 2 h; **b**: MeI, K_2_CO_3_, CH_3_CN, r.t., 4 h; **c**: AcOH, Br_2_, 60 °C overnight; **d**: NaBH_4_, MeOH, −20 °C to r.t., 3 h; **e**: TsCl, Et_3_N, CH_3_CN, reflux overnight; **f**: BBr_3_, CH_2_Cl_2_, 0 °C to r.t., 3 h.

The abundance of BDDE in the fresh red algae is very low (about 3 × 10^−3^% W/W) [12], which makes further investigation difficult. In order to obtain sufficient quantities of pure BDDE, we initiated development of an improved synthesis efforts on BDDE. The synthetic route established, shown in Scheme 1, was different from the published synthetic methods [18]. Although this route is four steps longer, with a total yield of 4.7%, the workup process is convenient and this route avoids the instability of the intermediate products and also avoids the production of byproduct (2-bromo-4,5-dihydroxybenzyl)(2,3-dibromo-4,5-dihydroxybenzyl) ether, which might complicate the purification process. Therefore, it is an alternate efficient approach to obtain the target compound. The structure of target compound **7** was confirmed by the following ^1^H NMR spectroscopic analysis and compared with the data in published literature [12,15]: ^1^H NMR (400 MHz, DMSO) δ: 4.344 (4H, s, H-7, 7′), 6.939 (2H, s, H-6, 6′), 9.567 (2H, s, HO-5, 5′), 10.085 (2H, s, HO-4, 4′). Compound **7** exhibited inhibitory activity against α-glucosidase with an IC_50_ value of 0.116 μM, which was consistent with BDDE isolated from marine algae [12,13,15]. Therefore, we established a valid synthesis route and provided enough amount of BDDE for the following studies both *in vitro* and *in vivo*, and also for the evaluations of its other bioactivities.

### 2.2. BDDE Binding Quenched the Intrinsic Fluorescence of α-Glucosidase

**Figure 2:**
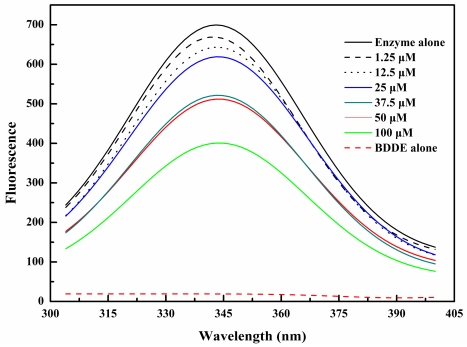
Intrinsic fluorescence spectra changes of α-glucosidase by BDDE. The enzyme (2 μM) was incubated with specified concentrations of BDDE (0–100 μM) for 30 min at 37 °C. The excitation wavelength was 295 nm and emission spectra were acquired by scanning from 300 to 400 nm.

The previously reported competitive inhibition on α-glucosidase *in vitro* [15] suggests that BDDE may directly bind to the active site of the enzyme. To test this hypothesis, we first investigated the BDDE induced changes in intrinsic tryptophan fluorescence of the α-glucosidase enzymes. As shown in Figure 2, when excitated at 295 nm, BDDE had no obvious fluorescence emission, and contributed negligible florescence interference. With the treatment of BDDE (0–100 μM), fluorescence intensity of α-glucosidase was quenched gradually in a concentration dependent manner. These results established that there is an interaction between BDDE and α-glucosidase, which induces a microenvironment variation for Trp residues in α-glucosidase. Possibly, this variation hinders the active centre formation and/or the binding of the substrates. The intrinsic fluorescence changes in α-glucosidase are similar to those in lysozyme induced by bromophenol blue [19], and many other enzymatic inhibitors could also induce fluorescence quenching of their target enzymes [20,21].

### 2.3. BDDE Binding Reduced the Hydrophobicity of α-Glucosidase

**Figure 3:**
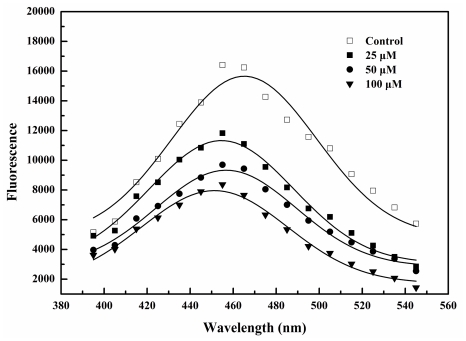
Changes of α-glucosidase-bis-ANS complex fluorescence by BDDE. α-Glucosidase (2 μM) was incubated for 30 min at 37 °C in the absence or presence of specified concentrations of BDDE (0–100 μM). Bis-ANS (15 μM) was then added, and fluorescence was measured after 15 min of incubation at 37 °C (excitation at 400 nm, emission at 395–545 nm) using a Cary Eclipse fluorescence spectrophotometer.

The hydrophobic characterization and environmental sensitivity of bis-8-anilinonaphthalene-1-sulfonate (bis-ANS) allow it to be widely used in measurement of protein surface hydrophobicity [22]. To study the BDDE induced changes on the hydrophobicity of α-glucosidase, bis-ANS was employed and the relative fluorescence intensities were shown in Figure 3. With increased concentrations of BDDE, the enzyme-bis-ANS fluorescence was reduced in a concentration-dependent manner. These results suggested that BDDE could reduce the hydrophobic surface of α-glucosidase. In our previous study, reduced enzyme hydrophobicity were also observed when α-glucosidase bound to another inhibitor, butyl-isobutyl-phthalate (BIP) [23]. Such inhibitor induced decreasing in the hydrophobicity supports the notion that poor hydrophobic surface usually hinder the formation of active center in the enzyme molecules.

### 2.4. BDDE Binding Influenced the Secondary Structures of α-Glucosidase

**Figure 4:**
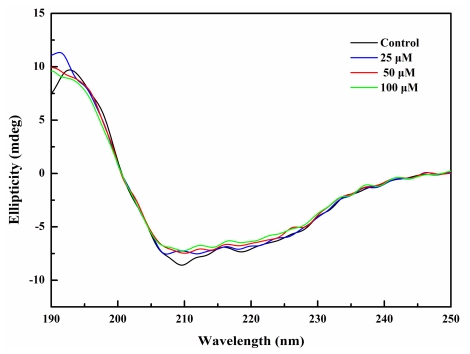
CD spectra of α-glucosidase in the absence or presence of BDDE. α-glucosidase (2 μM, dissolved in 20 mM potassium phosphate buffer, pH 6.8) alone or incubated with BDDE (0–100 μM) at 37 °C for 30 min. Spectra were acquired from 190 to 250 nm.

To study the effect of BDDE on the secondary structure of α-glucosidase, we measured the CD spectra (190–250 nm) of α-glucosidase. As shown in Figure 4, BDDE (0–100 μM) triggered a minor change in the CD spectrum of α-glucosidase, by increasing the α-helix (from 27.5% to 31.2%) and decreasing the β-sheet content (from 39.4% to 31.6%, Table 2). This finding further supports the proposition that BDDE binds to the enzyme and slightly changes the secondary conformation of α-glucosidase. The increase in α-helix induced by BDDE is similar to α-glucosidase inhibitor penicillamine, which also induces an increase in the α-helix content [24]. However, BDDE-induced secondary structure changes are in contrast to other α-glucosidase inhibitors, for example, curcuminoids analogs [7], BIP [23], and hydroxycoumarin derivatives [25], all of which decrease the α-helix content in the enzyme molecule. The reason that these inhibitors induce contrary changes in the α-helix content remains unknown. Nonetheless, it is clear that small conformational changes that hamper active center formation or prevent substrates binding will inactivate the enzyme.

### 2.5. Protein 3D Structure Generation and Molecular Docking

**Figure 5:**
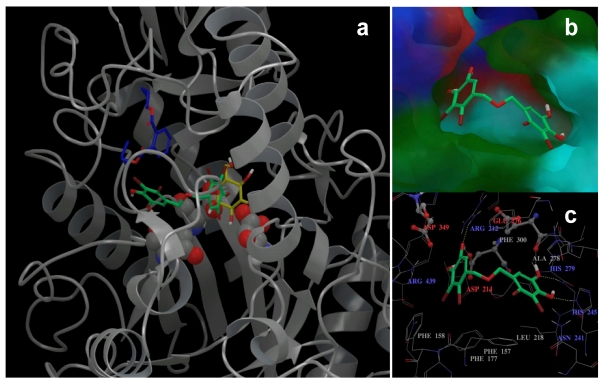
Structure model of BDDE-α-glucosidase complex. (**a**) Comparisons between docked BDDE (green-carbon), BIP (blue-carbon) and the crystal structure of glucose (yellow-carbon). Glucosidase is shown in grey ribbon, ligands are shown in solid sticks, the triad residues (Asp214, Glu276 and Asp349) are shown in a CPK model; (**b**) A close-up view of BDDE binding pocket. Molecular surface are shown and colored according to their residue properties (red: negatively charged; blue: positively charged; cyan: polar; green: hydrophobic); (**c**) The interactions between docked BDDE and nearby residues. The triad residues are emphasized in a ball-and-stick model, and hydrogen bonds are represented in dashed yellow lines.

Since the 3D structure of α-glucosidase is still unavailable, a homology model was developed based on the crystal structure of *S. cerevisiae* oligo-1,6-glucosidase (Figure 5a). To explore the potential protein ligand interactions, induced fit docking protocol was employed to dock BDDE into the glucose binding site of the non-cognate homology structure. The BDDE molecule was successfully docked into the active site with a highly favorable binding energy of −11.54 kcal/mol. The docked BDDE molecule was completely buried in the α-glucosidase binding pocket with part of the molecule reaching the catalytic center comprised of Asp214, Glu276 and Asp349 and overlapping with the position of glucose in its complex crystal structure, which adequately explains the competitive nature of BDDE; the remaining part of the molecule extended towards the protein surface. Using the same modeling and docking protocols, we have previously studied BIP, a non-competitive α-glucosidase inhibitor, and identified a novel ligand binding site located ~3 Å away from the catalytic center and close to the protein surface [23]. Direct comparison of the docking results of BDDE and BIP through structural alignment showed that there was no overlapping between the BIP and BDDE binding site, which indicates potential non-competitive binding between these two ligands. Detailed structural analysis (Figure 5b,c) suggested that the binding pocket of BDDE consisted of three areas: (1) a charged area which is located near the active center and consists of charged residues including the negatively charged catalytic triads and positively charged Arg439 and Arg212; (2) a polar area near protein surface that consists of residue His279, His235 and Asn241; and (3) a hydrophobic area sandwiched between residue Phe300, Ala278 and residue Phe158, Phe177, Phe157, Leu218. While the hydroxyl groups at the two ends of BDDE molecule formed multiple hydrogen bonds separately with residues in the charged and polar areas, the rest of the molecule were stabilized by hydrophobic interactions with nearby residues. The observed multiple hydrogen bonds formed by the hydroxyl groups of BDDE are consistent with the conclusion from previous studies that phenol moiety and hydroxyl groups in hydroxycoumarin derivatives are critical for their inhibitory activities [25]. The identified charged-hydrophobic-polar (C-H-P) binding pocket, which fits very well with the symmetrical feature of BDDE, provides structural insight for design and development of novel α-glucosidase inhibitors. However, further X-ray or NMR data are needed to verify this enzyme-inhibitor binding.

## 3. Experimental Section

### 3.1. Materials

The baker’s yeast α-glucosidase (EC 3.2.1.20), p-nitrophenyl glycosides, and bis-8-anilinonaphthalene-1-sulfonate (bis-ANS) were purchased from Sigma-Aldrich Chemical (St. Louis, MO, USA). Other reagents were reagent grade available and purchased from J&K Chemical Ltd. (Beijing, China).

### 3.2. Synthesis of BDDE

Procedures for the Preparation of Compounds **1** to **7**

#### 3-Bromo-4-hydroxy-5-methoxybenzaldehyde (2)

To a solution of compound **1** (15.2 g) in AcOH, Br_2_ (7 mL) was added at r.t. This mixture was stirred at r.t. for 2 h, and the compound **1** disappeared by TLC. Then the mixture was poured into cool water, and after filtration, the product compound **2** was obtained as white solid (20.7 g, yield: 90%).

#### 3-Bromo-4,5-dimethoxybenzaldehyde (3)

To a solution of compound **2** (20.7 g) in CH_3_CN, K_2_CO_3_ (19 g) and MeI (19 g) were added at r.t. Then this mixture was stirred at r.t. for 4 h. The mixture was poured into water and white precipitation was showed and the compound **3** was obtained after filtration as white solid (20 g, yield: 92 %).

#### 2,3-Dibromo-4,5-dimethoxybenzaldehyde (4)

Br_2_ (7 mL) was added to a solution of compound **3** (20 g) in AcOH at r.t. This mixture was stirred at 60 °C overnight. The TLC showed the compound **3** was consumed completely. Then the mixture was poured into cool water, and after filtration, the product compound **4** was obtained as white solid (19.3 g, yield: 73%).

#### (2,3-Dibromo-4,5-dimethoxyphenyl)methanol (5)

To a solution of compound **4** (19.0 g) in MeOH, NaBH_4_ (4.52 g) was added at −20 °C. Then the mixture was warmed to r.t. slowly and stirred for 3 h. After removing the solvent, the product compound **5** (13.3 g, yield: 70%) was obtained by washing with water.

#### 5,5′-Oxybis(methylene)bis(3,4-dibromo-1,2-dimethoxybenzene) (6)

To a solution of compound **5** (13 g) in CH_3_CN, 4.54 g of TsCl and 2.41 g of Et_3_N were added at r.t. Then the mixture was refluxing overnight. The TLC showed that compound **5** was completely consumed. The mixture was poured into cool water, after filtration, the product compound **6** was obtained as white solid (6.56 g, yield: 52%).

#### Bis(2,3-dibromo-4,5-dihydroxybenzyl) ether (7)

To a solution of compound **6** (6.0 g) in dry CH_2_Cl_2_, BBr_4_/CH_2_Cl_2_ (2.0 M) solvent (20 mL) was added at 0 °C. The mixture was warmed to r.t. slowly and stirred for 4 h. Then, cool MeOH was added to quench the reaction. After removing the solvent, the residue was separated by biotage® Flash chromatography (35% MeOH/H_2_O) to obtain compound **7** (1.2 g, yield: 21.8%).

### 3.3. Intrinsic Fluorescence Measurements

α-Glucosidase (2 μM) was pretreated with certain concentrations of BDDE (0–100 μM) for 30 min at 37 °C. The intrinsic fluorescence spectra (300–400 nm) were measured using a Cary Eclipse fluorescence spectrophotometer (Varian Inc. USA) with the excitation wavelength was 295 nm.

### 3.4. Hydrophobic Analysis of α-Glucosidase Using Bis-ANS

α-Glucosidase (2 μM) was incubated at 37 °C for 30 min in the absence or presence of certain concentrations of BDDE (0–100 μM). Bis-ANS (15 μM) was then added, and fluorescence was measured after incubation at 37 °C for 15 min (λ_ex_ = 400 nm, λ_em_ = 395–545 nm) using FlexStation II microplate spectrofluorometer (Molecular Devices, USA).

### 3.5. Circular Dichroism Spectroscopy

CD spectra (190–250 nm) of α-glucosidase (2 μM) treated with different concentrations of BDDE (0–100 μM) were measured with a JASCO 715 spectropolarimeter (JASCO, Tokyo, Japan). The spectra were collected and corrected by subtraction of a blank containing 20 mM potassium phosphate buffer (pH 6.8), reduction of noise, and smoothing. The program JWSSE (JASCO) was used for estimation of the secondary structure percentage of α-glucosidase based on the method of Yang *et al.* [26].

### 3.6. Molecular Modeling and Docking

Molecular modeling and docking studies were performed following the similar procedure as in our previous study [23]. Briefly, a 3D homology model of *Saccharomyces cerevisiae* α-glucosidase was built based on the complex crystal structure of α-d-glucose bound oligo-1,6-glucosidase (PDB ID: 3A4A) which shares 72% identical and 85% similar sequence with α-glucosidase. Sequence alignment and structural model building were performed using the protein structure prediction suite Prime (Schrödinger, New York, NY, USA). The constructed homology model was further optimized using the protein preparation wizard of maestro 9.0 with OPLS2001 force field [27]. Docking studies were then performed on the optimized model using the Induced-Fit-Docking (IFD) workflow [28], which is capable of sampling dramatic side-chain conformational changes as well as minor changes in the backbone structure. Specifically, the center of glucose molecule in the crystal structure was selected as the centriod for ligand-docking, residues within 6 Å distance of glucose were set to be flexible. IFD protocol was applied with default parameters to sample energetically reasonable side chains and to eliminate conformations with steric crashes. The docked protein-ligand complexes were ranked according to their IFD score made up of the protein-ligand interaction energy (GlideScore) and the protein molecular mechanics energy (Prime energy) in an implicit solvent model [28].

## 4. Conclusions

In summary, BDDE has been synthesized and its interaction with α-glucosidase was studied. Our results suggest that BDDE bind the enzyme molecule via both hydrogen-bonds and hydrophobic interactions. BDDE binding hindered the substrate from accessing the catalytic site and induced conformational changes on α-glucosidase, characterized by intrinsic fluorescence quenching, hydrophobicity reduction, and α-helix content increase. This study provided structural insights regarding the interaction between α-glucosidase and BDDE, and implied that BDDE could be considered as novel template for the design of α-glucosidase inhibitors.

## Figures and Tables

**Table 1:** IC_50_ values and *S. cerevisiae* α-glucosidase inhibition modes of bromophenols purified from different marine algae.

Marine bromophenol	(μM)	Inhibition mode	Reference
Bis(2,3,6-tribromo-4,5-dihydroxybenzyl) ether	0.03	Unknown	[13]
Bis(2,3-dibromo-4,5-dihydroxybenzyl) ether	0.098	Competitive	[12,13,15]
2,3,6-Tribromo-4,5-dihydroxybenzyl alcohol	11	Mixed	[15]
4-Bromo-2,3-dihydroxy-6-hydroxymethylphenyl-2,5-dibromo-6-hydroxy-3-hydroxymethylphenyl ether	25	Unknown	[12]
2,4,6-Tribromophenol	60.3	Mixed	[14]
2,3-Dibromo-4,5-dihydroxybenzyl alcohol	89	Mixed	[12]
3-Bromo-4,5-dihydroxybenzyl alcohol	100	Mixed	[13]
2,4-Dibromophenol	110.4	Mixed	[14]

**Table 2:** The secondary structure content of α-glucosidase influenced by BDDE.

BDDE (μM)	α-Helix (%)	β-Sheet (%)	β-Turn (%)	Random (%)
0	27.5	39.4	7.1	26
25	30.3	33.8	5.6	30.3
50	28.9	34.7	8.3	28.2
100	31.2	31.6	7.1	30.2

The secondary structure content of α-glucosidase is predicted by program JWSSE (JASCO).
